# Interleukin-1β and anaphylatoxins exert a synergistic effect on NGF expression by astrocytes

**DOI:** 10.1186/1742-2094-3-8

**Published:** 2006-04-04

**Authors:** Anne-christine Jauneau, Alexander Ischenko, Alexandra Chatagner, Magalie Benard, Philippe Chan, Marie-therese Schouft, Christine Patte, Hubert Vaudry, Marc Fontaine

**Affiliations:** 1 Institut Fédératif de Recherche Multidisciplinaire sur les Peptides n°23, INSERM U413, Faculté des Sciences, 76130 Mont-St-Aignan, France; 2 Research Institute of Highly Pure Biopreparations, St Petersburg, Russia

## Abstract

C3a and C5a anaphylatoxins are proinflammatory polypeptides released during complement activation. They exert their biological activities through interaction with two G protein-coupled receptors named C3aR and C5aR, respectively. In the brain, these receptors are expressed on glial cells, and some recent data have suggested that anaphylatoxins could mediate neuroprotection. In this study, we used RT-PCR and ribonuclease protection assays (RPA) to investigate the role of anaphylatoxins on neurotrophin expression by the human glioblastoma cell line T98G and by rat astrocytes. Our data show that for both cell types, anaphylatoxins upregulate expression of NGF mRNA. This response depended on a G protein-coupled pathway since pre-treatment of cells with pertussis toxin (PTX) completely blocked NGF mRNA increases. This effect was anaphylatoxin-specific since pre-incubation with anti-C3a or anti-C5aR antibodies abolished the effects of C3a and C5a, respectively. The regulation of NGF mRNA by anaphylatoxins was not accompanied by translation into protein expression, but there was a significant synergic effect of anaphylatoxins/IL-1b costimulation. Our demonstration of involvement of anaphylatoxins in the NGF release process by astrocytes suggests that C3a and C5a could modulate neuronal survival in the CNS.

## Introduction

Injury in the CNS produces a multi-faceted, complex cascade of events that includes immunological changes such as activation of the complement system and generation of antibodies, release of pro-inflammatory cytokines and chemokines, and production of reactive oxygen species leading to oxidative stress. Activation of the complement (C) system leads to release of various fragments among which the anaphylatoxins, C3a and C5a, are two proinflammatory polypeptides. C3a and C5a, which are liberated through cleavage of C3 and C5 by C convertases, exert their biological activities by binding to two G protein-coupled receptors named C3aR and C5aR, respectively [1]. There is evidence that C biosynthesis occurs in the CNS and all components of the C system can be synthesized locally by astrocytes, microglia and neurons [2]. Complement functions to eliminate intruding pathogens. However, there is now considerable evidence that increased complement synthesis and uncontrolled complement activation in the CNS contribute to pathological changes in the brain. Intrathecal complement activation has been shown to occur in multiple sclerosis, Alzheimer's disease, bacterial meningitis, stroke and other brain diseases [3,4]. Inflammatory reactions in these disorders are also associated with expression of pro-inflammatory cytokines, including IL-1β, TNF-α, IL-6, IFN-γ and IL-8. Excess expression of these cytokines can result in the destruction of the body's own cells, particularly neurons.

Several classes of neurons rely on neurotrophic factors, including nerve growth factor (NGF), for their survival and maintenance of function. Neurotrophins have many important physiological roles during and after CNS development [5]. Moreover, in brain disorders such as Alzheimer's disease increasing levels of endogenous NGF may be beneficial [6,7]. NGF is produced predominantly by neurons under normal physiological conditions; whereas astrocytes become the major site of NGF synthesis in the CNS during periods of rapid glial proliferation or after injury in the adult brain, [8-11]. Previous studies have shown that NGF secretion from astrocytes is modulated by various factors including glial cell growth, neurotransmetters and cytokines [12-14]. IL-1β is one of the most potent stimulators of NGF secretion in cultured neonatal astrocytes [15,16,14]. In normal, healthy brain, expression of IL-1β and its mRNA are very low [17], but these increase markedly in response to local inflammation, injury, or disease states such as Alzheimer's disease and stroke [18-21].

There is now growing evidence that complement, and more specifically the anaphylatoxins, could participate in neuroprotection in the brain [22-24]. To further examine the potential roles of C3a and C5a in the CNS, we examined the release of NGF by astrocytes upon stimulation with anaphylatoxins, which thus may participate to neuroprotection.

## Materials and methods

### Reagents, cytokines and antibodies

PTX, human recombinant C5a, and IL-1β were purchased from Sigma, St Quentin Fallavier, France. Anti-C3a monoclonal antibody (G10) and anti-C5aR antibody, used to block the effect of C3a and C5aR, respectively, have been characterized previously [25,26].

Human C3a was generated by activation of C, and purified as previously described [25].

Multiple-Associated-Peptide (MAP)-C3a and (MAP)-C5a peptides, corresponding to the C-terminal part of the anaphylatoxins (amino acids correspond 64–77 for C3a and 61–74 for C5a, respectively), attached to a poly-lysine comb (eight peptidic monomers) were synthesized by solid phase synthesis (Applied Biosystem) and were purified by reverse phase HPLC. Sequences were ascertained by amino acid analysis. The concentrations of MAP peptides were calculated with the whole Molecular Mass of the MAP peptide.

### Cell culture

The human glioblastoma cell line T98G was obtained from American Type Culture Collection (Rockville, MD, USA). These cells were screened routinely using a Mycoplasma Detection Kit (Boehringer Mannheim, Meylan, France) to ensure that they were mycoplasma free. Cells were grown in Ham's F12 culture medium (Biowhittaker, Emerainville, France) supplemented with 1% penicillin and streptomycin (Life Technologies, Cergy-Pontoise, France), and 10% heat-inactivated fetal calf serum (Life Technologies).

Primary astrocytes were prepared from brain of new-born rats and cultivated as previously described [27]. All stimulations with anaphylatoxins or IL-1β were realized in medium without serum (Ultradoma, Biowhittaker). The astrocyte marker glial fibrillary acidic protein (GFAP) was detected by flow cytometry in 95–97% of these cells. CR3-positive cells were not detected in primary astrocyte cultures using the OX42 monoclonal antibody (ECACC, Sigma) and analysis by flow cytometry.

### RNA extraction

Total RNA was extracted from cells using a guanidium isothyocyanate method followed by ultracentrifugation onto a cesium chloride cushion. Total RNA (50 μg) was treated for 20 min at 37°C with 90 U of RQ-1 RNase- free DNase (Promega, Charbonnières, France) in 100 μl of buffer (40 mM Tris-HCl pH 8, 10 mM NaCl, 6 mM MgCl_2 _and 10 mM CaCl_2_) and 200 U of RNasin ribonuclease inhibitor (Promega) to remove all traces of contaminating genomic DNA.

### PCR primers

Human NGF and glyceraldehyde 3-phosphate deshydrogenase (GAPDH) primers were chosen according to their cDNA sequences reported in EMBL Data Library under Accession Numbers X52599 and M33197. Their sequences from 5' to 3' were as followed: NGF sense [AGG TGC ATA GCG TAA TGT CC], NGF antisense [CCT TGA CAA AGG TGT GAG TC], GAPDH sense [TGC CAT CAA CGA CCC CTT CA] and GAPDH antisense [TGA CCT TGC CCA CAG CCT TG]. The theoretical size was 642 pb for NGF and 549 pb for GAPDH.

### RT-PCR

Reverse transcription was carried out for 60 min at 37°C in 30 μl (final volume) with 2 μg of total RNA, 20 U RNasin (Promega), 250 pmol random hexamer primers pd(N)6 (Pharmacia Biotech, Orsay, France), 1 mM dNTPs (Pharmacia), 5 mM DTT and 400 U Moloney Murine Leukemia Virus Reverse Transcriptase (MMLV)-RT (Life Technologies) in the reaction buffer (250 mM Tris-HCl, 375 mM KCl, 15 mM MgCl_2_). The reaction mixture was then heated to inactivate the MMLV-RT. PCR was carried out with 5 μl of cDNA pool, in 50 μl final volume with 1.5 mM MgCl_2_, 200 μM de (dNTPs), 100 pmol of NGF primers and 2 U of Taq DNA polymerase (Life Technologies) in the reaction buffer (20 mM Tris-HCl, 0.1 mM d'EDTA, 1 mM DTT). The PCR steps used were: denaturation for 4 min at 94°C, 25 to 30 cycles [denaturation 94°C for 40 s, annealing at 57°C for 50 s and extension at 72°C for 90 s], and final elongation step at 72°C for 10 min. PCR was performed in a Hybaid Omnigene thermocycler (Schleicher and Schuell, Céra-labo, Ecqueville, France. The absence of contaminant DNA was routinely checked by RT-PCR on negative control samples in which either the RNA samples were replaced with sterile water, or the MMLV-RT was omitted. For semi-quantitative RT-PCR, PCR was realized with a GAPDH:NGF primers ratio of 1:75 and 1 μCi [^33^P]dATP (Redivue, Amersham, Les Ulis, France). Experiments were conducted in which total RNA was amplified with different cycle numbers for GAPDH and NGF primers to assure that RNA bands after amplification were detected within the linear part of the amplifying curves. Autoradiograms were analyzed using Lecphor image analyzer (Biocom, Les Ulis, France). Results are expressed as a ratio of the area of the band of interest to the mean of the area of the housekeeping gene band. The NGF mRNA value, from unstimulated cells, was set to one unit arbitrarily and values for the other samples were calculated relative to this.

### Multiprobe RNase protection assay (RPA)

After total RNA isolation, RPA was performed using the RiboQuant Multiprobe RNase Protection Assay System (BD PharMingen, Le Pont de Claix, France), according to the manufacturer's instructions. Briefly, the provided rat neurotrophin template set (rNT-1) contained probes for six neurotrophins (NGF, brain-derived neurotrophic factor (BDNF), glial cell line-derived neurotrophic factor (GDNF), ciliary neurotrophic factor (CNTF), neurotrophin-3 (NT-3) and NT-4) and two housekeeping genes (GAPDH and L-32). To synthesize anti-sense cRNA, the probes were labeled with [^32^P]α UTP (800 Ci/mmol, 10 mCi/ml; Amersham) using a transcription kit according to the manufacturer's manual. Ten micrograms of each sample were used for hybridization with the anti-sense RNA probe at 56°C for 12–16 h, followed by digestion of free probe and unprotected ssRNA with RNase solution (RNase A plus RNase T1). The remaining dsRNA was then extracted in chloroform-isoamyl alcohol (50:1) and was precipitated with ethanol and separated on a 7 M urea/6% polyacrylamide gel. A part of the undigested probe was used as marker standard. After drying, the gel was placed in an exposure cassette with a phosphor screen for 48 h. Bands were detected by phosphorimaging using ImageQuant software (Molecular Dynamics). A standard curve plotted with the undigested probe markers was used to identify the bands of various genes in the experimental samples. Results are expressed as a ratio of the volume of the band of interest to the mean of the volumes of the bands for the housekeeping genes. The neurotrophin mRNA value, from unstimulated cells, was set to one unit arbitrarily and values for the other samples were calculated relative to this.

### Enzyme-linked immunosorbent assay (ELISA)

Concentrations of NGF protein in culture supernatant samples were determined by a specific sensitive ELISA (sensitivity 15.6 pg/ml): NGF E_max _Immunoassay System (Promega), according to manufacturer's instructions. The absorbance was measured at A_450 nm _with a plate reader (Labsystems iEMS Reader MF) and NGF content in the samples was determined by comparison with a NGF standard curve. The level of NGF in the culture supernatants was expressed as pg/ml per 10^6 ^cells.

### Statistical analysis

Data are expressed as mean ± SEM. Statistical analysis was performed using Student's t test. Differences were considered statistically significant at p < 0.05.

## Results

### C3a and C5a anaphylatoxins increase NGF mRNA expression in human glioblastoma cell line T98G

In a first approach, we studied NGF mRNA expression in the human glioblastoma cell line T98G. This cell line expresses C3a and C5a receptors [28,29] and has been shown to respond to anaphylatoxin stimulations, inducing IL-6 mRNA expression [30].

**Figure 1:**
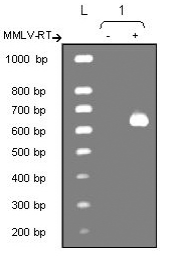
*Expression of NGF mRNA by unstimulated T98G cells analyzed by RT-PCR*. Lane L represents the size markers (100 bp ladder) and lane 1 the NGF amplicon (642 bp) with (+) or without (-) the MMLV-RT.

Expression of NGF mRNA was first analyzed in unstimulated T98G cells by RT-PCR, and PCR products were visualized by agarose gel electrophoresis and staining with ethidium bromide (Fig. 1). A unique 642 bp band was observed, corresponding to the expected size of NGF amplicon. Thus, unstimulated T98G cells constitutively expressed NGF mRNA. No amplification product appeared for the negative control where MMLV-RT was omitted.

**Figure 2:**
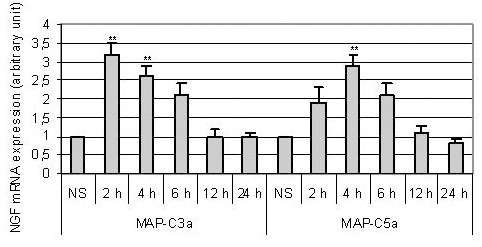
*Expression of NGF mRNA after stimulation of T98G celsl by MAP-C3a (**A**) or MAP-C5a (**B**) (10^-8^M)*. RNA was extracted after 2 h, 4 h, 6 h, 12 h and 24 h of stimulation and analyzed by semi-quantitative RT-PCR using GAPDH as internal standard. Bars represent the mean ± SEM for three independent experiments. **, p < 0.01, statistically significant compared with control as determined by Student's *t *test (NS = non stimulated cells).

**Figure 3:**
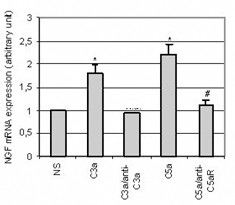
*Expression of NGF mRNA after stimulation of T98G cell line by C3a or C5a anaphylatoxins (10^-8^M)- Effect of pre-incubation with anti-C3a or anti-C5aR antibodies*. C3a anaphylatoxin was pre-incubated for 30 min with an anti-C3a antibody (diluted 1/100); or cells were pre-incubated for 30 min with an anti-C5aR antibody (diluted 1/100) before a 4 h stimulation by C3a or C5a (10^-8^M). Total RNA was extracted and RT-PCR was performed. Bars represent the mean ± SEM for three independent experiments. *, p < 0.05, statistically significant difference compared with non-stimulated cells (NS) as determined by Student's *t *test; #, p < 0.05, statistically significant difference compared with cells stimulated with C5a as determined by Student's *t *test.

T98G cells were then stimulated by MAP-C3a or MAP-C5a (10^-8^M), two strong peptidic anaphylatoxin analogs and total RNA was extracted after different stimulation times (2 h, 4 h, 6 h, 12 h and 24 h). NGF mRNA expression was then analyzed using semi-quantitative RT-PCR using GAPDH as internal standard. Results are presented as relative fold-increase over control (unstimulated cells) (Fig. 2). NGF mRNA expression was increased by 3.2 fold after 2 h of stimulation by MAP-C3a and by 2.9 fold after 4 h for MAP-C5a. Upregulation of NGF mRNA expression induced by MAP-C3a or MAP-C5a was also observed following stimulation using human purified C3a and recombinant C5a. T98G cells were stimulated with C3a or C5a (10^-8^M) and NGF mRNA expression was assessed by semi-quantitative RT-PCR. After 4 h of stimulation, NGF mRNA level was increased by 1.8 fold and 2.2 fold, respectively (Fig. 3).

### Anaphylatoxin-upregulated NGF mRNA expression is dose-dependent and specific

T98G cells were stimulated with MAP-peptides or anaphylatoxins in a range of 10^-13^M to 10^-7^M. Total RNA was extracted after 4 h of stimulation and NGF mRNA expression was measured. From 10^-10^M to 10^-8^M, we observed a linear relationship between the NGF mRNA expression and the concentration of the different agonists, showing dose-dependent increases in NGF mRNA (data not shown). A significant response was obtained when concentrations reached 10^-10 ^M for MAP-peptides and 10^-9^M for anaphylatoxins with comparable activities for C3a and C5a derivatives; the effect was maximum at 10^-8 ^M for both agonist types. These concentrations ranges match those recorded in previous studies on astrocytes [30,31].

**Figure 4:**
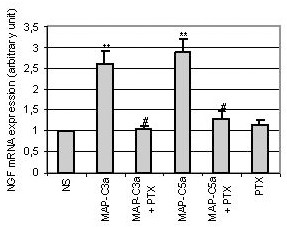
*Influence of toxin pertussis (PTX) on the NGF mRNA production by T98G cells following stimulation with MAP-C3a or MAP-C5a (10^-8^M)*. Cells were pre-incubated for 4 h with PTX (200 ng/ml) and then stimulated by MAP-C3a or MAP-C5a for 4 h. Cells were also incubated with PTX alone. Total RNA was extracted and RT-PCR was performed. Bars represent the mean ± SEM for three independent experiments.**, p < 0.01, statistically significant difference compared with non-stimulated cells (NS) as determined by Student's *t *test; #, p < 0.05, statistically significant difference compared with cells stimulated with MAP-peptides as determined by Student's *t *test.

In order to ascertain the specificity of anaphylatoxin upregulation of NGF mRNA, we tried to block their effect using specific antibodies. For this experiment, we used two antibodies that did not cross-react, anti-C3a did not inhibit the C5a effect and anti-C5aR did not modify the C3a effect (data not shown). Pre-incubation of C3a anaphylatoxin with an anti-C3a antibody (1μg/ml) during 30 min completely blocked the upregulation of NGF gene expression induced by C3a alone (Fig. 3). Similarly, when cells were pre-incubated during 30 min with an anti-C5aR antibody (1μg/ml) before C5a stimulation, the C5a-induced NGF mRNA upregulation was abolished (Fig. 3). C3a and C5a anaphylatoxins are known to bind to distinct receptors that are both functionally coupled to G proteins [32,33]. To confirm that MAP-peptide stimulation, leading to upregulation of NGF mRNA, acts through the G protein coupled receptor, T98G cells were pre-incubated with Pertussis toxin (PTX) (200 ng/ml) for 4 h prior to MAP-peptide stimulation. After 4 h of either stimulation by anaphylatoxin agonists or no stimulation, the level of NGF mRNA was quantified. PTX alone had no effect on the constitutive NGF expression, but pre-treatment of cells by PTX completely abrogated the upregulation of NGF mRNA expression induced by anaphylatoxin agonists (Fig. 4).

### MAP-C3a and MAP-C5a upregulate NGF mRNA expression by rat primary astrocytes

To confirm previous results obtained with T98G, we examined neurotrophin expression by rat primary astrocytes stimulated by MAP-C3a or MAP-C5a. Variation of neurotrophin expression was assessed by the sensitive RPA technique. In the same series of experiments, we determined NGF, BDNF, CNTF, GDNF, NT-3 and NT-4 mRNA expression.

**Figure 5:**
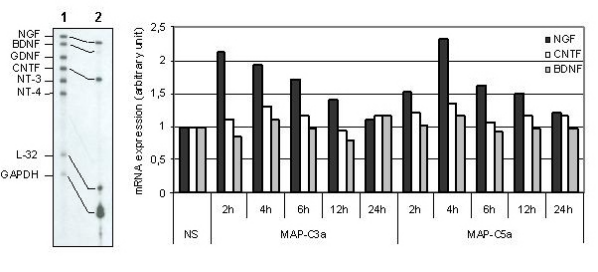
*Expression of neurotrophic factors by rat primary astrocytes stimulated by MAP-C3a or MAP-C5a (10-8M)*. Autoradiogramm of neurotrophin mRNA expression by unstimulated rat primary astrocytes is shown on the left panel: Lane 1 represents the Multi-Probe Template Set not treated with RNases and Lane 2 the neurotrophin mRNA expression by unstimulated rat primary astrocytes. Note that each probe band (Lane 1) migrates slower than its protected band (Lane 2); this is due to flanking sequences in the probe that are not protected by mRNA. RNA was then extracted after 2 h, 4 h, 6 h, 12 h and 24 h of stimulation and analyzed using L-32 and GAPDH as internal standards. On the right panel, results are represented as histograms and expressed as relative fold increase over control (NS = non-stimulated cells). This is a representative graph of n = 3 independent experiments.

We first observed that unstimulated rat astrocytes constitutively express NGF, BDNF and CNTF mRNA (Fig. 5*left*); GDNF, NT-3 and NT-4 mRNA were only detected on overexposed autoradiograms (not shown). Rat astrocytes were then stimulated by MAP-C3a or MAP-C5a (10^-8^M) and total RNA was extracted after different stimulation times (2 h, 4 h, 6 h, 12 h and 24 h). mRNA expression was then determined using GAPDH and L-32 as internal standards. Results are presented as relative fold-increase over control (unstimulated cells) (Fig. 5*right*). MAP-peptide stimulations of astrocytes induced an increase of NGF mRNA, multilplied by 2.1 after 2 h stimulation with MAP-C3a and by 2.3 after 4 h with MAP-C5a. MAP-peptide stimulation did not increase BDNF or CNTF mRNA and did not increase the level of expression of GDNF, NT-3 nor NT-4 mRNA. These experiments were repeated three times and were reproducible.

### Anaphylatoxin stimulation increased NGF secretion by T98G cell line

**Figure 6:**
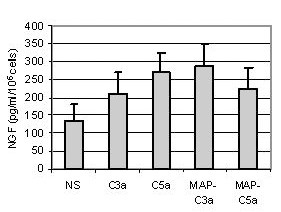
*Production of NGF by T98G cells analyzed by ELISA after incubation with C3a, C5a, MAP-C3a, MAP-C5a (10*^-8^*M) for 48 h*. The concentration of NGF was measured in the supernatants and was expressed as pg/ml per 10^6 ^cells. Bars represent the mean ± SEM for three independent experiments.

To confirm that increases in mRNA translate into NGF secretion, we performed ELISA. The supernatants of stimulated T98G cells were collected after 12 h, 24 h, 48 h and 72 h stimulation by either MAP-peptides or anaphylatoxins (10^-8^M) and the concentration of NGF protein was analyzed by specific sensitive ELISA. A basal level of NGF, produced by T98G cells, was observed (133 ± 47 pg/ml/10^6 ^cells) and anahylatoxins did not significantly increase this NGF secretion (Fig. 6). After 48 h of stimulation, NGF levels reached 212 ± 70 pg/ml/10^6 ^cells for C3a, 268 ± 56 pg/ml/10^6 ^cells for C5a, 287 ± 64 pg/ml/10^6 ^cells for MAP-C3a and 225 ± 68 pg/ml/10^6 ^cells for MAP-C5a. Although we observed constantly increased NGF concentrations in supernatants of stimulated cells, these increases were not statistically significant even at 48 h post-stimulation.

### Synergistic effect of anaphylatoxins and IL-1 beta on NGF secretion

**Figure 7:**
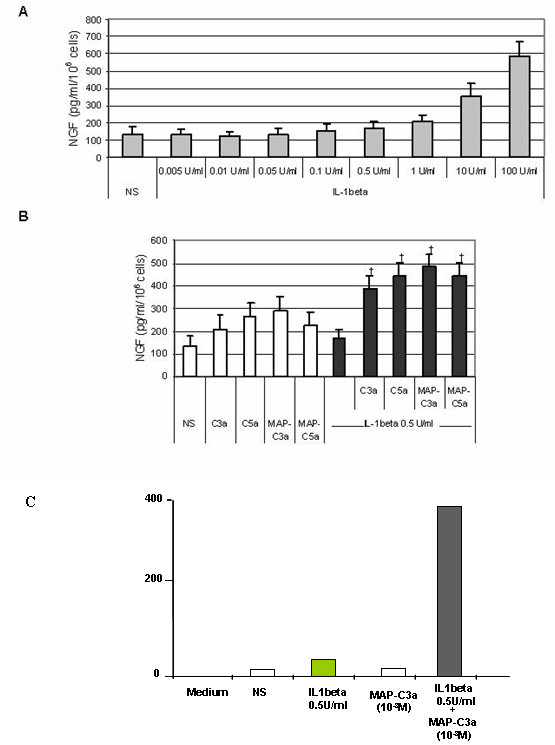
*Effect of anaphylatoxins/IL-1β co-stimulation on NGF release*. First, cells were stimulated for 48 h by a range of IL-1β concentrations to determine the maximal concentration of IL-1β that did not induce NGF release by T98G cells **(A)**. Second, T98G cells were co-stimulated for 48 h with sub-optimal dose of IL-1β (0.5 U/ml) as well as with C3a, C5a, MAP-C3a or MAP-C5a (10^-8^M) **(B)**. NGF release by rat astrocytes following MAP-C3a (10^-8^M) and IL-1β (0.5 U/ml) co-stimulation for 48 h is shown in (C). The concentration of NGF was measured in the supernatants by ELISA and was expressed as pg/ml per 10^6 ^cells. Bars represent the mean ± SEM for three independent experiments. +, p < 0.05, statistically significant compared with cells stimulated with anaphylatoxins or MAP-peptides alone as determined by Student's *t *test.

Anaphylatoxins are generated in an inflammatory context where cytokines are also released, especially IL-1β. Thus, we investigated the effects of anaphylatoxin/IL-1β co-stimulation on NGF release. First, we measured the release of NGF in supernatants of T98G cells after 48 h of stimulation by various doses of IL-1β in order to determine the maximum concentration of IL-1β that did not increase NGF secretion (Fig. 7A). We observed that concentrations of IL-1β below 0.5 U/ml did not enhance NGF secretion by T98G cells. Then, we co-stimulated T98G cells over 48 h with IL-1β (0.5 U/ml) and with C3a, C5a, MAP-C3a or MAP-C5a (10^-8^M). The resultant NGF levels are reported in Fig. 7B. Co-stimulation with IL-1β led to high increases of NGF secretion: from 170 ± 42 pg/ml/10^6 ^cells for IL-1β to 388 ± 56 pg/ml/10^6 ^cells for IL-1β +C3a, to 443 ± 60 pg/ml/10^6 ^cells for IL-1β +C5a, to 486 ± 54 pg/ml/10^6 ^cells for IL-1β +MAP-C3a and to 453 ± 57 pg/ml/10^6 ^cells for IL-1β +MAP-C5a. These results show a significant synergic effect of anaphylatoxin and IL-1β stimulation on NGF production. To confirm these results obtained with T98G cells, we examined NGF expression by rat primary astrocytes co-stimulated with MAP-peptides and IL-1β (0.5 U/ml/10^-8^M) over 48 h. As expected, MAP stimulation did not induce NGF release. but the MAP-peptides and IL-1β synergistically stimulated astrocyte NGF protein expression, as illustrated in figure 7C with MAP-C3a and IL-1β co-stimulation.

## Discussion

These experiments aimed at showing the potential role of anaphylatoxins to produce neurotrophins in order to protect neurons from injury. This hypothesis comes from the observation that C3a has neuroprotective effects against NMDA-induced neuronal death only in mixed cultures of neurons and astrocytes [34]. We postulated that this indirect effect of C3a on neuroprotection could be due, at least in part, to neurotrophin release by astrocytes.

It is well established that complement is produced in the brain, that complement activation permits the release of the anaphylatoxins, C3a and C5a; and that these signal through their respective receptors, C3aR and C5aR, since stimulation of glial cells by anaphylatoxins can increase cytokine production [24,30,31]. The roles of anaphylatoxins on brain cells remain ill-characterized and they may have functional roles independent of their classical role as mediators of inflammation. Altought complement has been implicated as a mediator of neuroinflammation and neurodegeneration, as in Alzheimer's disease [35,36], some inflammatory mediators, in particular the anaphylatoxins, are reported to have neuroprotective role.

In the present study, we sought to obtain further clues on the potential roles of C3a and C5a anaphylatoxins in neuroprotection by investigating the effects of C3a and C5a in parallel with their peptidic analogs, MAP-C3a and MAP-C5a on NGF release by astrocytes. Preliminary studies were performed by RT-PCR using the human T98G cell line. Our laboratory has previously shown that these cells express C3aR and C5aR, and using a cell line provided uswith homogenous material perfectly adapted for using RT-PCR technique. Thus, we observed that stimulation of T98G cells by anaphylatoxins induced increased expression of NGF mRNA. This upregulation was shown to be dose-dependent as NGF mRNA expression varied with the concentration of anaphylatoxins. Optimum concentrations (10^-8 ^M) are in agreement with previous studies [30,31]. Pre-treatment of cells with PTX completely blocked the effects of anaphylatoxinx and the MAP-peptides, suggestong that their effects were mediated through their specific receptors C3aR and C5aR. Pre-incubation with anti-C3a or anti-C5aR antibodies abolished the C3a- and C5a-induced NGF mRNA increase respectively, showing that the response was specific. Thus, the NGF mRNA increase acted *via *the anaphylatoxins/anaphylatoxin receptors and was not due to contaminants in anaphylatoxin preparations.

Analysis of NGF mRNA expression following anaphylatoxin stimulation was also studied using rat primary astrocytes analyzed by RPA. We showed that unstimulated rat astrocytes constitutively express NGF, BDNF and CNTF mRNA. Furthermore we observed a significant NGF mRNA increase after anaphylatoxin stimulations whereas BDNF and CNTF mRNA expression remained unchanged.

We consistently observed an increase in NGF concentation in cell supernatants following anaphylatoxin stimulation compared to unstimulated cells. It appeared that this effect was not statistically significant even for long incubation periods (48 h). In a previous study, we showed increased IL-8 mRNA expression [31] in response to anaphylatoxins, although this was not translated into secretion of cytokine. This IL-8 secretion was induced by co-stimulation of astrocytes with IL-1β and anaphylatoxins. A synergistic effect of IL-1β with TNF-α on NGF secretion by cultured rat astrocytes has been reported [37]. Since IL-1β has no effect on C3aR and C5aR expression by T98G cells [31] we investigated the effects of anaphylatoxins/IL-1β costimulation on NGF release. We demonstrate here significant synergetic effects of IL-1β and anaphylatoxin-induced NGF-release. The significant increase in NGF secretion is likely to be a result of synergistic actions of IL-1β and anaphylatoxins since this dose of IL-1β had no effect on its own. It was established previously that anaphylatoxins do not induce IL-1-β expression [30] ruling out the possibility that NGF upregulation by anaphylatoxins is due to an indirect effect via IL-1-β.

Our data, showing that anaphylatoxins in synergy with IL-1β stimulate astrocyte NGF secretion, are consistent with the hypothesis that many cell types release complement proteins as a protective mechanism against threats to cell viability. A previous study showed that C3a anaphylatoxin significantly induces NGF mRNA and protein production in human microglia [24]. This effect was obtained with a lower dose of C3a that that employed here. Moreover, in contrast to our results with astrocytes, C3a stimulation alone was sufficient to stimulate NGF secretion by microglia. In response to CNS injury, both microglia and astrocytes undergo structural and functional changes in a time-dependant manner. Microglia respond earlier than do astrocytes, and their response is often transient, whereas reactive astrocytes persist in their activated state. Thus, astrocytes may in a time-dependent manner supplant and continue the initial microglial NGF production. Anaphylatoxins may play a conditioning role in this process, allowing astrocytes to release neurotrophins in an inflammatory context.

## Conclusion

In contrast to studies showing C5a-induced apoptosis in a human neuroblastoma cell line in vitro [38,39], recent publications have implicated anaphylatoxins in neuroprotection [40,22]. Thus, mice genetically deficient in complement component C5 are more susceptible to kainic acid excitotoxicity than are normal mice [41,42]. Moreover, other studies have recently shown that C5a can directly protect neurons against glutamate neurotoxicity [23] and that C3a has protective effects against NMDA-induced neuronal death [34], suggesting involvement of anaphylatoxins in neuroprotection. Our findings elucidate a molecular basis for such anaphylatoxin-mediated neuroprotection, and fit well with the general concept that anaphylatoxins released locally are involved in tissue repair or remodelling [43].

## Abbreviations

CNS: central nervous system, NGF: nerve growth factor; PTX: pertussis toxin, RPA: ribonuclease protection assay.

## Competing interests

The author(s) declare that they have no competing interests.

## Authors' contributions

AC, J carried out all the experiments and contributed to the writing of the manuscript.

AI synthesised the MAP-peptides.

AC helped for experimentation and drafted the manuscript and responded to rewievers.

MB carried out the co-stimulation experiments on rat astrocytes.

PC helped to experimentation.

CP and MT, S performed culture cell.

HV and MF are the supervisors of the laboratory.

All authors read and approved the final manuscript.
